# Psychosocial factors associated with speech perception in older adult cochlear implant users: a scoping review

**DOI:** 10.3389/fnins.2025.1636354

**Published:** 2025-09-10

**Authors:** Ilona Anderson, Katharina Angerer-Klaunzer, Jane Opie

**Affiliations:** MED-EL GmbH, Innsbruck, Austria

**Keywords:** older adult, psychosocial performance, loneliness, quality of life, significant other, family support, caregiver burden

## Abstract

**Background:**

The population of older adults (OAs) is significantly increasing, and with that is the reality of OAs having hearing loss (HL). Although there is no hearing screening for adults, some OAs do consult their audiologist or otologist and receive a cochlear implant (CI). There are several studies showing that CI surgery in OAs is safe, and post-CI speech perception is similar to younger adults. However, there is little knowledge about what psychosocial factors may be associated with speech perception outcomes in OAs. The objective of this scoping review is to address these open questions.

**Methods:**

This scoping review was conducted in line with the Preferred Reporting Items for Systematic Reviews and Meta-Analyses (PRISMA) guidelines. PubMed and the Cochrane Library were searched for studies in English and German published between Jan 2020 and Jan 2025 reporting on psychosocial factors relating to speech perception outcomes in OAs with CIs.

**Results:**

6/94 studies met the inclusion criteria. The six included publications considered the associations of quality of life, frailty, depression, and family support on speech perception in OAs with CIs. The association between these factors and sound perception was variable.

**Conclusion:**

Although CIs are not uncommon as a treatment for HL in OAs, few studies have addressed the associations of psychosocial factors with speech perception in this population, or vice versa. More research needs to be conducted to determine these psychosocial factors and their impact on speech perception to better care for OAs with CIs.

## 1 Introduction

Cochlear implants (CIs) are small electronic devices that help provide hearing to individuals with severe-to-profound hearing loss who do not benefit from hearing aids (HAs). A CI consists of an external part placed behind the ear and an internal part that is surgically placed under the skin (NIH, 2024).^1^ CIs provide objective and subjective benefit to people with hearing loss (HL) of all ages. In particular, good speech perception abilities enable conversational communication in quiet with one person and with many persons, as well as in noisy situations even when the background noise is competing speech. Despite this remarkable success, speech perception performance still considerably varies among CI users, perhaps more so in older adults (OAs). OAs are defined as adults aged 60 years or older by the World Health Organization (WHO) (United Nations, n.d.). Existing literature shows that duration of severe-to-profound HL and device programming are two well-known factors associated with speech perception: shorter duration of hearing loss and a well-programmed audio processor generally support better outcomes.

Psychosocial factors, such as depression, loneliness, family support, social integration, wellbeing, frailty, and quality of life (QoL) might also have a relation with speech perception and positive outcomes in OAs and might account for individual variability. Hearing researchers have studied relations between psychosocial outcomes and speech perception and self-reported benefit. It is important to do so because auditory communication is deeply embedded in the lives of OAs; it is essential to maximize outcomes along both auditory and non-auditory dimensions.

With regard to HAs, Mothemela et al. (2023) investigated associations between social networks, self-reported mental health, QoL, work situation, and self-reported HA outcomes. Results in 398 mostly OAs revealed that all psychosocial factors positively correlated with the hearing outcomes. Interestingly, social networks that specifically involved other HA users were important.

Hickson et al. (2014) also studied associations between psychosocial factors and successful HA use in two groups of HA users older than 60 years: one group of well-performing HA users and one group of poorly performing HA users, based on the users’ self-reported HA use and benefit. Influencing factors found in the well-performing group were greater support from significant others, more positive attitudes towards HAs, and a greater sense of self-efficacy with HA handling. Similarly, Dwarakanath and Manjula (2022) examined attitudes towards HL and personality as non-audiological factors associated with HA outcomes and found that people with a more positive attitude towards HL and with more extroverted personality traits reported more subjective HA benefit and participated more in social interactions.

Brewster K. K. et al. (2022) examined which individual characteristics of OAs with HL and depression determined the extent of their benefit from HA treatment. People with poorer pre-operative self-perceived hearing disability, speech recognition, physical performance, and language functioning experienced the greatest alleviation of their depression symptoms after a period of HA use. Hearing-related quality of life (HRQoL) is an important non-audiological psychosocial factor that is studied in relation to HA use. Understanding HRQoL not only provides insight into the multifaceted benefits of hearing devices but also allows comparisons to be made with other similar and dissimilar medical treatments. In addition, it is an important metric for health technology assessment and reimbursement. Wolff et al. (2024) investigated correlations between HA use and HRQoL using a metric sensitive to changes in auditory performance. The questionnaire was applied to 1894 OAs with a mean age of over 60 years, who were either new HA users (*n* = 1,362) or experienced HA users (*n* = 532). Data analyses revealed that self-reported benefit, speech perception, and hours of HA use positively correlated with better HRQoL. Using a disease-specific measure of HRQoL that measures self-perceived disability, Ahn et al. (2023) looked across a spectrum of routinely measured audiological abilities to determine which had the greatest impact on self-reported hearing disability. The findings suggest that it is speech perception that most impacts hearing disability in OA HA users with listeners with better speech perception scores experiencing the least self-perceived disability.

In summary, the HA literature has shown that non-audiological psychosocial factors and speech perception are complex concepts that interact with each other in a multi-dimensional manner. Understanding these associations allows hearing researchers and hearing healthcare professionals better understand OA HA users. As an extension to OAs with severe-to-profound HL, the present paper aims to examine, via a scoping review, non-audiological psychosocial factors related to speech perception in OAs with CIs.

## 2 Methods

### 2.1 Search criteria

A systematic literature search was performed following the Preferred Reporting Items for Systematic Reviews and Meta-analyses (PRISMA) guidelines for scoping reviews (PRISMA-ScR) (Tricco et al., 2018). This scoping review aimed to identify relations among various psychosocial factors and between psychosocial factors and speech perception outcomes in OAs with HL treated with CIs.

PubMed and the Cochrane Library were searched in April 2025 for articles published in English or German between Jan 2020 and Jan 2025. A detailed search strategy was developed according to the population, intervention, comparison, outcome (PICO) criteria using combinations and variations of search terms related to the population (e.g., “older adults,” “elderly,” “senior”; “hearing loss,” “deafness,” “presbycusis”), outcome (e.g., “hearing outcome,” “speech perception,” “hearing performance”), and intervention (e.g., “cochlear implant”) in scope (subject headings and MeSH terms). No comparator was chosen as the scope of the review was restricted to psychosocial factors in relation to speech perception in CI users. The detailed search strategy is given in Table 1. Backwards citation mining of relevant included articles was performed.

**Table 1:** Combinations of search terms for psychosocial factors and speech perception in OAs with CIs for PubMed and the Cochrane Library.

Search step	Search terms	Term category	PICO
1	“Older adults” OR “elderly” OR “geriatric” OR “aging population” OR “senior” OR “seniors”	Indication/population: older adults	P
2	Hearing loss OR hearing impairment OR deafness OR deaf OR “loss of hearing” OR “age-related hearing loss” OR “age related hearing loss” OR presbycusis OR “severe-to-profound” OR “severe to profound”	Indication/population: severe-to-profound HL	P
3	“Hearing outcome” OR “hearing performance” OR “speech performance” OR “speech recognition” OR “speech perception” OR “speech discrimination” OR “speech” OR “word recognition” OR “word perception” OR “word discrimination” OR “sentence test”	Outcome: speech perception/hearing outcomes	O
	Cochlear implant	Intervention: cochlear implantation	I
4	1 AND 2 AND 3 AND 4	Population + outcome: hearing outcomes of older adults with severe-to-profound HL	P + I + O
5	Limit to *Language*: English or German		P + I + O
6	Limit to *Publication date*: 01 Jan 2020 to 31 Jan 2025		P + I + O

### 2.2 Selection criteria

Titles and abstracts were screened by one author (KA-K), then two authors (IA, JO) independently assessed the full text of articles included based on title and abstract and performed data extraction. Study authors were not contacted when study information was unclear or not reported. Disagreements regarding the inclusion of a study were discussed between the two authors (IA and JO) to reach a mutual consensus.

Original articles reporting on psychosocial factors, such as QoL, loneliness, family situation/support, social integration and wellbeing outcomes in OAs (aged ≥60 years) with HL treated with CIs were included. All articles had to have included speech perception outcomes. All speech perception outcomes, irrespective of the type of test (e.g., words, sentences, testing in quiet or noise, testing conditions) and test language, were considered relevant. Studies on mixed populations of younger adults and OAs were included if results for OAs were reported separately.

Exclusion criteria included: (1) pre-clinical studies; (2) age of study population unclear or <60 years, or results for OAs not reported separately; (3) no speech perception outcomes reported; (4) study population did not include CI users; (5) no relevant psychosocial factors affecting speech perception outcomes addressed; (6) result of (ongoing) study not available; (7) systematic review/meta-analysis.

Risk of bias of the included studies was assessed using the Joanna Brigg’s Critical Appraisal Checklist for Case Series and Cohort Studies (Moola et al., 2015).

### 2.3 Data extraction

Two authors (IA, JO) independently performed data extraction. Data extracted from studies included the geographical region of the study; sample details (e.g., number of participants; degree of HL/pre-operative pure tone audiogram, if reported); study design and follow-up; reported outcome measures; reported post-CI speech perception performance, and any reported correlations with post-CI speech perception.

## 3 Results

### 3.1 Results of the literature search

Eighty-nine studies were retrieved from PubMed, four from Cochrane, and five through citation mining. After removal of duplicates and ongoing Cochrane trials with no published results, 94 studies were included for title and abstract screening. This screening excluded 85 articles. Full-text review of the remaining nine articles excluded three articles, resulting in six articles being included in the final review. One of the included studies was published outside of the reporting period (Tang et al., 2017). Of the six publications one publication was a prospective study, four were cross-sectional, and one was retrospective. The six included articles reported information on audiological outcomes, QoL, frailty, neurocognitive measures, co-morbidities, depression, and family support. None of the retrieved articles reported on (social) integration or social inclusion. The three articles excluded during full-text review did not categorize the population into OAs and younger adults.

**Figure 1:**
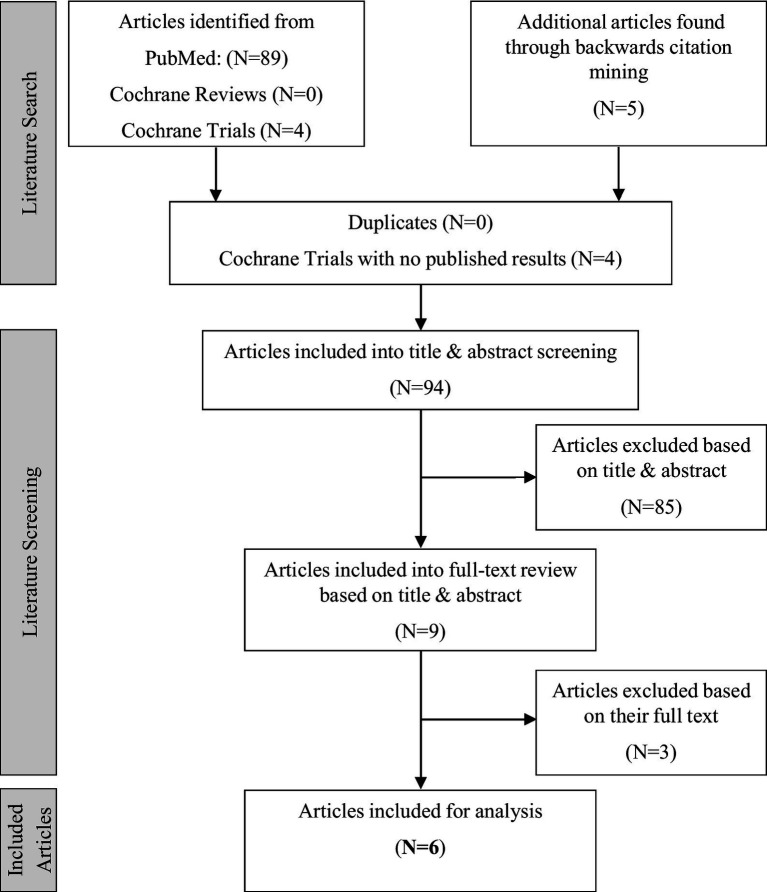
PRISMA flow chart of the literature search conducted for the scoping review of psychosocial factors and speech perception outcomes in OAs with CIs.

The search process is summarized in Figure 1. The details of the studies are given in Table 2. Data provided include geographical region, sample inclusion, study design, follow-up time, outcome measures, reported performance, and statistical results.

**Table 2:** Data extraction table for the scoping review on psychosocial factors affecting speech perception in OAs with CIs.

Reference	Geographical region	Sample details	Follow-up	Outcome measures	Reported performance
(Aylward et al., 2022)**—**frailty and quality of life after cochlear implantation in older adults	USA	*N* = 143 older adults ≥65 years Mean age at CI: 76.1 years (SD, 7.1) Mean pre-CI PTA: 91.8 dB HL (SD, 15.4)	NA—cross-sectional study with retrospective chart review	Demographics PTA, speech recognition (CNC words, AzBio sentences, HINT sentences) QoL (modified version of the NCIQ; adapted for post-lingually deafened older adults) 11-point frailty index (FI)	No significant correlation between patient age and FI. *Audiological outcomes* post-CI: mean PTA: 37 dB (SD, 15.2); mean CNC words score: 49.7% (SD, 24.3%); mean AzBio sentence score: 46.5% (SD, 29.7%); mean HINT sentence score: 77.1% (SD, 22.5%); No significant correlation between FI and audiologic outcomes. *QoL*: Higher QoL correlated with lower FI (better health) and larger improvement in PTA post-CI. Higher scores in QoL domains of activity limitation and social interaction correlated with lower FI.
(Imagawa et al., 2024)—generic and hearing-specific QoL in older adult CI users	Japan	*N* = 18 older adults (≥60 years); mean age 72.7 years (SD, 6.96); All with bilateral severe HL (≥90 dB HL) or moderate-to-severe HL (70–90 dB HL) with ≤50% speech perception in the best-aided condition	NA—cross-sectional study at 1–5 years post-CI with retrospective chart review (post-CI outcomes at 6 months post-CI);	Speech perception: CI2004 (Japanese CI speech perception test battery including consonants, monosyllables, words and sentences in quiet) HHIE THI DHI HUI-3	*Demographics*: Majority lived with family members 11.1% employed *Audiological outcomes*: median scores at 6 months post-CI: monosyllables: 40%; words: 72%; sentences: 77%; *QoL* at mean 3.2 years post-CI: HHIE: 52.0 (severe handicap range), range: 18–74; THI: 14.0 (no handicap range); DHI: 0.0 (no handicap range); HUI-3 hearing: 0.74; HUI-3 utility: 0.63 Correlation analysis: No correlation of HUI-3 sub-scores with HHIE, THI or DHI. Moderate significant correlation between HUI-3 and duration of HL. No correlation between HUI-3 and speech perception. Strong significant correlation between HUI-3 and HrQoL. No correlation between HUI-3 generic utility and other HUI-3 sub-scores. Longer duration of HL pre-CI was associated with larger improvements of generic QoL post-CI. Reduced HUI-3 sub-domain of hearing was associated with a lower overall generic QoL. Analysis of individual profiles for poor QoL cases (HUI-3 utility score <0.5): Case 2: Low utility function score (0.70), severe handicap in HHIE (54); lower score for emotion (0.77) and pain (0.88), influenced by restrictions in economic and leisure activities and lumbar spinal stenosis; Case 6: Very long-term HL (>60 years); low hearing utility function score (0.70); severe handicap in HHIE (58); speech difficulties, due to chronic subdural hematoma (speech utility score 0.76); Case 8: low hearing utility function score (0.70); severe handicap in HHIE (74); poor speech perception (subjective and objective); low HUI-3 scores for speech (0.76), cognition (0.74) and pain (0.88) Case 17: low hearing utility (0.54) despite mild–moderate handicap in HHIE (30), good speech perception (76% for words, 77% for sentences) due to issues with face-to-face conversation and conversation with multiple people in HUI3;
(Sorrentino et al., 2020)—cognitive function and quality of life in adult patients with cochlear implants	Italy	*N* = 25 older adult CI users (≥65 years); median age at CI: 71 years *N* = 19 young adult CI users (≤50 years); median age at CI: 39 years *N* = 25 NH older adults (control group); Profound progressive post-lingual deaf- ness	Min. 12 months post-CI Longitudinal analysis: cross sectional analysis comparing chart review from pre-CI to the last examination	PTA, speech perception: word recognition, sentence recognition (derived from Test Abilità Uditive) GBI MMSE	*Audiological outcomes*: Significant improvement from pre-CI to 12 months post-CI, and from pre-CI to last examination in all speech perception tests. Median word recognition scores: OA: pre-CI 5%, 12 months post-CI 70%, last examination 75%; median sentence recognition scores: OA: pre-CI 0%, 12 months post-CI 90%, last examination 75%. *QoL*: Mean GBI scores post-CI: OA: overall benefit 39.9 (range: −55 to +78), general domain 46.5, social benefit 50.0, physical health 1.4. Correlation analysis: Significant positive correlations between speech perception and GBI scores in older adults for: GBI overall benefit, GBI general health, GBI physical health.
(Tang et al., 2017)—rehabilitation and psychological determinants of cochlear implant outcomes in older adults	USA	*N* = 55 older adults (≥65 years) (*N* = 33 with full data); mean age at CI: 75.8 years (range: 65–87 years) All with post-lingual severe-to-profound SNHL	12 months Prospective	Speech perception: AzBio sentences GBI Trait EI (emotional intelligence) questionnaire General health (Charles co-morbidity index)	*Audiological outcomes*: Significant improvement of AzBio sentence scores by +47% post-CI. No significant effect of age at testing, duration of HL or general health (CCI scores). 78.8% of patients used their CIs for >12 h/day. Device usage did not affect speech perception scores. Use of tablet computers was associated with significantly higher speech perception post-CI (+18% AzBio scores for tablet users compared to non-tablet users). Cohabitation positively correlated with post-CI speech perception (+22% in AzBio scores compared to people who lived alone). Speech therapy/rehabilitation did not affect post-CI speech perception. *QoL*: Significant improvement of mean GBI scores by +38.2 post-CI. No association between post-CI GBI score and duration of HL. No association between post-CI GBI scores and post-CI speech perception. Device usage did not affect post-CI GBI scores. Technology use did not affect post-CI GBI scores. Speech therapy/rehabilitation positively affected post-CI GBI scores (+15.8 in GBI scores post-CI for patients who attended at least the mandatory consultation compared to those who did not attend any rehabilitation session). Cohabitation did not affect post-CI GBI scores. *Other factors*: EI scores did not affect speech perception or QoL post-CI. EI scores did not affect adherence to speech therapy/rehabilitation.
(Tokat et al., 2021)—quality of life in older adults with cochlear implantation: Can it be equal to that of healthy older adults?	Turkey	*N* = 54 older adults (>65 years) with CIs; mean age at CI: 71.32 years (SD, 1.6) Mean pre-CI PTA4 108.7 dB HL *N* = 54 NH older adults (control group); mean age 70.3 years (SD, 1.8)	12 months Retrospective review	PTA, speech perception (Turkish monosyllabic words) WHOQOL-BREF WHOQOL-OLD GDS	*Audiological outcomes*: post-CI: Significant improvement in in CI group compared to pre-CI; Mean PTA4 in CI group: 33.1 dB HL; mean Turkish monosyllable score in quiet: 75.7%; *QoL*: Mean WHOQOL-BREF scores: CI users: 2.91 NH controls: 2.96; No significant differences between CI and NH groups in WHOQOL-OLD and WHOQOL-BREF. WHOQOL and WHOQOL-BREF increased in parallel with speech perception improvement. Significant correlation of speech perception with sub-domains of social participants, social relations and psychological domains. Significant correlation of WHOQOL-OLD and duration of CI use. Longer duration of CI use was associated with higher QoL sub-domains of sensory abilities and past, present and future activities. Lower age was associated with sub-domains of autonomy and social participation. Older age was associated with sub-domain death and dying. Changes in GDS score were not significant
(Völter et al., 2020)—benefits of cochlear implantation in middle-aged and older adults	Germany	*N* = 30 middle-aged (MA) adults (50–64 years); mean age 57.33 years (SD, 4.48) *N* = 41 older adults (OA) (≥65 years); mean age 72.33 years (SD, 5.27) All with bilateral severe-to-profound HL (mean pre-CI PTA in better hearing ear: MA 79.33 dB; OA 79.23 dB HL)	12 months	Speech perception [Freiburger monosyllables (FMS) in quiet; OLSA in noise] NCIQ AlaCog GDS-15	*Audiological outcomes*: Significant improvement post-CI compared to pre-CI; Mean post-CI FMS scores in quiet: OA 56.33%; mean OLSA in noise: OA + 1.25 dB *QoL*: Significant improvement in NCIQ scores post-CI; Mean NCIQ scores: OA: pre-CI 50.55; post-CI 66.42; Correlation analysis (OA): OA with better improvement in speech perception post-CI had better scores in NCIQ sub-domain self-esteem and advanced sound perception. OA with shorter duration of HL had larger improvement in NCIQ sub-domain advanced sound perception post-CI. *Comorbidities: depression* (GDS-15) Higher pre-CI GDS-15 depressive scores was associated with worse post-CI AlaCog cognition results. Post-CI depression was predictive for post-CI NCIQ sub-domain physical sound perception.

AlaCog, Alzheimer’s disease cooperative study cognitive subscale; AzBio, Arizona Biomedical Sentences; CCI, Charles comorbidity index; CI, cochlear implant; CI2004, Japanese cochlear implant speech perception test battery; CNC, consonant-nucleus-consonant; DHI, dizziness handicap inventory; dB, decibel; dB HL, decibel hearing level; EI, emotional intelligence; FI, frailty index; FMS, Freiburger monosyllables; GBI, Glasgow Benefit Inventory; GDS, geriatric depression scale; GDS-15, 15-item geriatric depression scale; HHIE, hearing handicap inventory for the elderly; HL, hearing loss; HINT, hearing in noise test; HrQoL, health-related quality of life; HUI-3, Health Utilities Index Mark 3; MA, middle-aged; MMSE, mini-mental state examination; N, number of participants; NA, not applicable; NCIQ, Nijmegen cochlear implant questionnaire; NH, normal hearing; OA, older adults; OLSA, Oldenburg Sentence Test; PTA, pure tone average; PTA4, four-frequency pure tone average; QoL, quality of life; SD, standard deviation; SNHL, sensorineural hearing loss; THI, tinnitus handicap inventory; WHOQOL-BREF, World Health Organization Quality of Life-Brief Version; WHOQOL-OLD, World Health Organization Quality of Life-Older Adults Version.

### 3.2 Risk of bias assessment

The publications chosen reported on patient populations from five different countries: two from the USA, one from Germany, one from Italy, one from Turkey, and one from Japan, which reflects an unbiased geographical approach. Table 3 shows risk of bias across the included publications (Moola et al., 2015; NIH, 2025). Reported data show minimal risk of bias.

**Table 3:** Risk of bias assessment of the included publications.

Risk of bias	Aylward et al. (2022)	Völter et al. (2020)	Tokat et al. (2021)	Imagawa et al. (2024)	Sorrentino et al. (2020)	Tang et al. (2017)
Were the criteria for inclusion clearly defined?	y	y	u	u	y	y
Were the study subjects and the setting described in detail?	y	y	y	u	y	y
Was a control used?	n	y	y	n	y	n
Was the exposure measured in a valid and reliable way?	y	y	u	y	y	y
Were objective, standard criteria used for measurement of the condition?	y	y	y	y	y	y
Were confounding factors identified?	n	n	n	n	n	n
Were strategies to deal with confounding factors identified?	n/a	n/a	n/a	n/a	n/a	n/a
Were the outcomes measured in a valid and reliable way?	u	y	y	y	y	y
Was appropriate statistical analysis used?	y	y	y	y	y	y
Was ethics approval sought?	y	y	y	y	y	y
Did the authors acknowledge conflicts of interest?	y	y	y	y	y	y

y, yes; u, unclear; n, no; n/a, not applicable.

### 3.3 Audiological outcomes

All included publications reported on audiological outcomes, which included pure tone audiograms and speech tests (monosyllables, sentences in quiet and/or sentences in noise). In all cases, significant improvement was observed at either 6 months (Imagawa et al., 2024) or 1 year post CI (Tang et al., 2017; Sorrentino et al., 2020; Völter et al., 2020; Tokat et al., 2021; Aylward et al., 2022).

### 3.4 Quality of life

Each of the reviewed publications reported correlations between better speech perception and higher scores on some measures of QoL.

The Nijmegen Cochlear Implant Questionnaire (NCIQ) (Hinderink et al., 2000) is a disease-specific QoL questionnaire. OAs with better scores on the NCIQ sub-domains ‘self-esteem’ and ‘advanced sound perception’ had better speech perception (Völter et al., 2020). OAs with a shorter duration of HL also had a larger improvement in the sub-domain ‘advanced sound perception’.

Imagawa et al. (2024) correlated the Hearing Handicap Inventory for the Elderly (HHIE) (Ventry and Weinstein, 1982), a disease-specific questionnaire measuring social and emotional well-being, with the Health Utility Index Mark 3 (HUI-3), a generic QoL questionnaire (Feeny et al., 2002) and showed that there was no correlation between the HUI-3 sub-scores and the HHIE. However, there was a strong correlation between the HUI-3 and HHIE total scores. There was moderate, yet significant correlation between the HUI-3 and duration of HL, with longer duration of HL showing larger improvement in generic QoL post CI; but no correlation between HUI-3 and speech perception scores.

The WHO Quality of Life Brief Version (WHOQOL-BREF) (WHO, 1998) and the WHO Quality of Life Older Adults Module (WHOQOL-OLD) (Power et al., 2005) were measured on 54 OAs (Tokat et al., 2021). Both measures increased in parallel with speech perception improvement and those who had used the CIs longer had higher QoL scores on the sub-domains ‘sensory abilities’ and ‘past, present, and future activities’. There was a significant correlation of speech perception with ‘social relations’ and ‘psychological’ domains. The younger OA groups associated more with ‘autonomy’ and ‘social participation’, while the older OA groups associated more with ‘death and dying’.

OA patients measured on the Glasgow Benefit Inventory (GBI) (Robinson et al., 1996) showed a positive correlation between speech perception scores and ‘overall benefit’, ‘general health’, and ‘physical health’ (Sorrentino et al., 2020). In contrast, Tang et al. (2017) showed no correlation between GBI and post-operative speech perception or duration of HL.

### 3.5 Loneliness and frailty

Frailty may be a factor of social integration. Patients with higher QoL scores measured by the NCIQ and lower Frailty Index (FI) scores showed improved post-operative pure tone audiogram scores. Patients with better QoL scores in ‘activity limitation’ and ‘social limitation’ had lower FI scores (Aylward et al., 2022).

Depression links into the search term ‘loneliness’. Higher depression scores measured by the Geriatric Depression Scale (GDS-15) (Yesavage et al., 1983) were predictive for post-CI NCIQ scores in the sub-domain ‘physical sound perception’, which would reflect on speech perception outcomes (Völter et al., 2020). In another study considering depression, no significant changes were seen on the GDS; there was a significant positive correlation with all domains of the WHOQOL-BREF and WHOQOL-OLD (Tokat et al., 2021).

### 3.6 Family and caregivers

Two publications considered the impact of family or care givers on the outcome of speech perception. In one publication, 88.9% of OAs lived with family (Imagawa et al., 2024), but this was not correlated with any outcomes. In the other publication, co-habitation positively correlated with improved post-operative speech perception compared to those who lived alone (Tang et al., 2017).

## 4 Discussion

The aim of this scoping review is to report on published data on psychosocial factors associated with hearing outcomes in OAs using CIs. Specifically, this review provides an update on published literature relevant to the effect of psychosocial performance, including (social) integration, social inclusion, wellbeing, loneliness, QoL and significant other, family support and caregiver burden on speech perception outcomes of OAs (aged ≥60 years) with HL treated with CIs. The reviewed literature showed that there were impacts on speech perception outcomes, but the literature did not test causality or direction of effect, and this should be considered throughout this discussion. It also reflects the multi-factorial dimensions within this scoping review.

QoL is the most reported psychosocial factor, discussed by 6/6 of the included articles. WHO defines QoL as “an individual’s perception of their position in life in the context of the culture and value systems in which they live and in relation to their goals, expectations, standards, and concerns” (WHO, 2025). Within the realm of HL, psychosocial issues exist due to communication impairment and social issues. Disease-specific QoL measures are one way of evaluating the impact of communication and social issues relative to the effectiveness of treatment procedures for HL (Lailach et al., 2024).

All the reported papers reported on QoL as it is seen as an important outcome measure in CI users, showing this is an important part of why OAs receive a CI. Essentially, assessing QoL allows clinicians to understand a patient’s subjective experience (Dorismond et al., 2023), particularly in OAs to whom QoL may be more important than speech outcomes. Some publications used generic measures of QoL. The WHOQoL-BREF and WHOQoL-OLD showed a correlation between speech perception and social participation, social relations, and psychological domains (Tokat et al., 2021). The GBI was used in two publications. One showed a correlation between improved speech perception and overall benefit, general health, and physical health (Sorrentino et al., 2020), while the other showed no effect at all (Tang et al., 2017). The same was seen using the HUI-3 as a measure of QoL. Although the HUI-3 sub-domain of ‘hearing’ was associated with lower generic QoL, there was no correlation between the HUI-3 and speech perception outcomes (Imagawa et al., 2024). Despite the mixed outcomes, the reported results showed that generic QoL questionnaires may not be sensitive to the impact that QoL may have on speech perception outcomes.

Two papers used the disease-specific NCIQ questionnaire in their studies. The NCIQ sub-domains of ‘self-esteem’ and ‘advanced sound perception’ correlated with improved speech perception, but the NCIQ total score did not (Völter et al., 2020) while in the other study, NCIQ sub-domains of ‘activity limitations’ and ‘social interaction’ correlated with better frailty scores, but not with speech perception outcomes (Aylward et al., 2022). In a meta-analysis of CI-specific QoL measures, data showed a weak positive correlation with speech perception, which is in line with the data shown for OAs in this scoping review. The outcome of the meta-analysis showed that, although QoL improves post-CI, it is a poor predictor of speech perception outcomes (McRackan et al., 2018).

Aylward et al. (2022) considered QoL and frailty; frailty was measured by the 11-factor modified FI (Velanovich et al., 2013). Frailty is seen as an accurate metric in predicting post-operative morbidity, and although not widely used in the field of otolaryngology, it might be a useful metric to consider for determining health status (Aylward et al., 2022; Yuen et al., 2023). A surgeon may see a person as a ‘frail patient’ (Velanovich et al., 2013) and one may assume that most ‘frail patients’ would be OAs. Aylward et al. (2022) found no correlation between frailty and audiological outcomes. In a study on adults of all different age groups, those with better health had better QoL and better audiological performance (Maron, 2025), while in another study, frailty was not associated with speech perception, but those with mild frailty had better speech perception outcomes (Yuen et al., 2023). Fostering prevention, such as adult hearing screening, early fitting of HAs, and early CI provision, may likely improve QoL. The literature clearly showed that people with lower FI had better QoL.

Besides frailty, other comorbidities, such as depression, may impact speech perception outcomes in OAs (Völter et al., 2020). Depression includes sadness, feelings of low self-worth or guilt, a loss of interest in daily activities, and disturbed appetite or sleep (WHO, 2025). Both Völter et al. (2020) and Tokat et al. (2021) measured depression using the GDS (Yesavage et al., 1983) and showed that CI use did not improve depression. In fact, Völter et al. (2020) also mentioned that duration of HL did not correlate with mental health either. This lack of change is also seen in CI users of all age groups (Lailach et al., 2024; McIlhiney et al., 2025). The data of McIlhiney et al. (2025) showed that depression worsened after 12 months of device use across adults of all ages. Lack of change was also seen in studies on OAs wearing HAs (Lawrence et al., 2020; Brewster K. et al., 2022). HL may be associated with an increase in social and emotional loneliness (Pronk et al., 2014) which may lead to depression in OAs (West, 2017). However, there were no specific measures of loneliness in this scoping review.

Most of the OAs in the study by Imagawa et al. (2024) lived with family, which may have an impact on outcomes in general—improving social well-being, QoL, and speech perception outcomes. Co-habitation positively correlated with post-CI speech perception (22% higher scores compared to those who lived alone) (Tang et al., 2017). This is affirmed by a study including 130 OA wearing HAs. Here, OA living arrangements were defined by type of facility where they lived and co-habitation. Data shows that those who were in assisted living had lower speech perception than those who were not (Francis et al., 2015). They suggested this was because of less independence, not the same amount of rehabilitation, and less social and moral support. A lack of social support may result in less conversational stimuli which are essential to maintain and/or improve speech perception outcomes. The clinician should encourage family and/or social support to motivate the patient over time (Sorrentino et al., 2020).

## 5 Conclusion

This scoping review revealed psychosocial factors that may be associated with hearing outcomes in OAs. Six included publications showed significant correlations between speech perception outcomes and QoL. Depression had a negative impact on speech perception; family support showed positive effects. The scoping review failed to find specific literature on (social) integration, social inclusion, and loneliness. This highlights the need to do further research on these important factors that may benefit OAs to see how they can improve QoL and listening skills. Further research and understanding of psychosocial issues are needed for caring for OAs who may need more support and care; professionals should see these factors as clinically relevant in their practice.

## Footnotes

^1^ https://www.nidcd.nih.gov/health/cochlear-implants
